# Axitinib Reverses Resistance to Anti-Programmed Cell Death-1 Therapy in a Patient With Renal Cell Carcinoma

**DOI:** 10.3389/fimmu.2021.728750

**Published:** 2021-10-26

**Authors:** Yonghao Yang, Hao Huang, Tiepeng Li, Quanli Gao, Yongping Song, Zibing Wang

**Affiliations:** Department of Immunotherapy, Affiliated Cancer Hospital of Zhengzhou University & Henan Cancer Hospital, Zhengzhou, China

**Keywords:** anti-angiogenic, PD-1, immunotherapy, low-dose, resistance, axitinib

## Abstract

Owing to broad and notable clinical anti-tumor activity, anti-programmed cell death-1 (PD-1)/anti-programmed cell death-ligand 1 (PD-L1) antibodies have been indicated for almost all types of cancer, and form a part of the current standard of care. However, a large proportion of patients do not respond to anti-PD-1/PD-L1 therapy (primary resistance), and responders often develop progressive disease (acquired resistance). The mechanisms of resistance are complex and largely unknown; therefore, overcoming resistance remains clinically challenging, and data on reversing anti-PD-1 resistance are scarce. Herein, we report the case of a 58-year-old woman with renal cell carcinoma associated with Xp11.2 translocation/transcription factor E3 gene fusion, who had already showed resistance to both anti-PD-1 monotherapy and standard-dose axitinib. However, she finally achieved a partial response with a continuous combination therapy comprising low-dose axitinib and anti-PD-1. We speculate that axitinib played a key role in reversing the primary resistance to anti-PD-1 therapy. Interestingly, we observed that the number of peripheral regulatory T cells increased after the standard-dose axitinib therapy, with accompanied tumor enlargement; however, after the dose was reduced, the number of regulatory T cells decreased gradually, and the tumor regressed. We also reviewed relevant literature, which supported the fact that low-dose axitinib might be more beneficial than standard-dose axitinib in assisting immunotherapy. Given that this is a single-case report, the immunomodulatory effect of axitinib requires further investigation.

## Introduction

There have been considerable advances in cancer immunotherapy in recent years, these are mainly due to in-depth studies on the programmed cell death protein 1 (PD-1)/programmed cell death-ligand 1 (PD-L1) pathway. Binding of PD-1 to its ligands on tumor cells suppresses T cells through a negative feedback loop, thereby leading to immune response evasion (1, 2). Antibodies targeting PD-1 or PD-L1 can block this inhibitory signaling pathway and reactivate the anti-tumor immune response. Presently, based on clinical data from many phase III clinical trials, an anti-PD-1 or anti-PD-L1 agent is part of the standard of care for most types of advanced malignancies (3).

Despite the broad and notable anti-tumor activity of anti-PD-1/PD-L1 antibodies, a large proportion of patients still do not respond to anti-PD-1/PD-L1 therapy (primary resistance), and the patients who respond to them would often develop tumor progression again (acquired resistance). With the rapid expansion of the population treated with anti-PD-1/PD-L1 antibodies, the issue of resistance to anti-PD-1 therapy is of increasing concern. The mechanisms of resistance are complex and largely unknown; therefore, overcoming resistance remains clinically challenging (4, 5), and data on reversing anti-PD-1 resistance are scarce. Herein, we present a case in which axitinib successfully reversed primary resistance to anti-PD-1 therapy in a patient with renal cell carcinoma (RCC) associated with Xp11.2 translocation/transcription factor E3 gene fusion (Xp11.2/TFE3). We also reviewed relevant literature on the subject.

## Case Description

**Figure 1:**
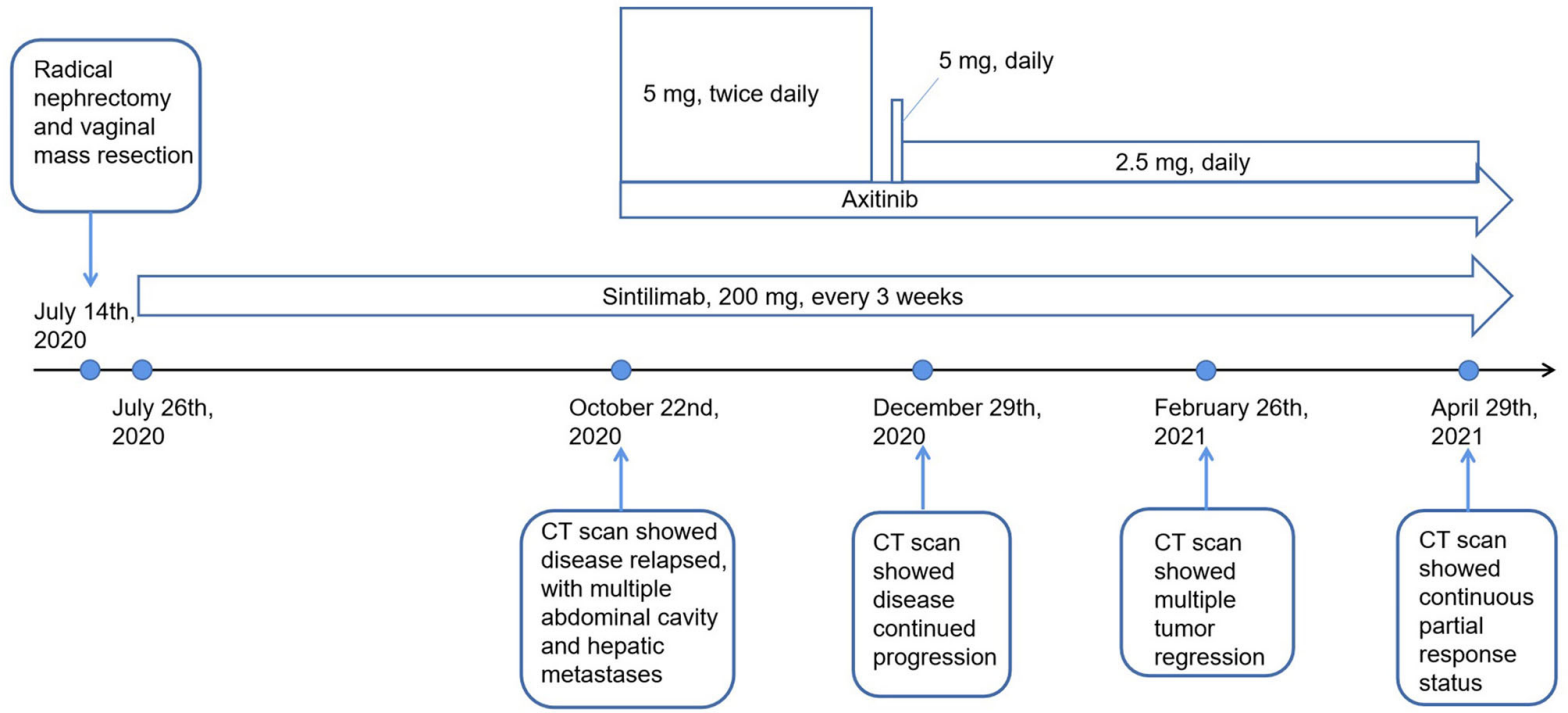
Flow chart of patient’s main management process.

The patient’s treatment process is shown in **Figure 1**. A 58-year-old woman presented to our hospital in early July 2020 with pain in the left flank region, gross hematuria, and minor vaginal bleeding. Before this time, she was in good health and had no history of chronic diseases, such as hypertension, diabetes, and coronary heart disease. She was allergic to cephalosporin antibiotics, characterized by rashes. Enhanced whole-body computed tomography (CT) revealed an 8.6 × 7.2 × 6.5 cm left renal mass with obvious enhancement, which we highly suspected was a left renal carcinoma. Simultaneously, pelvic magnetic resonance imaging revealed a 7 × 7 mm nodule at the vaginal end. She underwent laparoscopic radical nephrectomy and vaginal mass resection on July 14, 2020. Results of the pathological investigations of specimens from the two sites were consistent for RCC associated with Xp11.2/TFE3, which is a rare type of RCC in adults and a pathology with a poor prognosis (6). There is currently no standard treatment for this rare type of RCC; therefore, an intra-departmental consultation was convened. This patient already had vaginal metastasis, which reflected the aggressive nature of the RCC. In the end, we agreed to start postoperative adjuvant treatment with sintilimab, which is an anti-PD-1 antibody that has been developed and approved in China. From July 26, 2020, our patient received sintilimab (200 mg) every 3 weeks.

**Figure 2:**
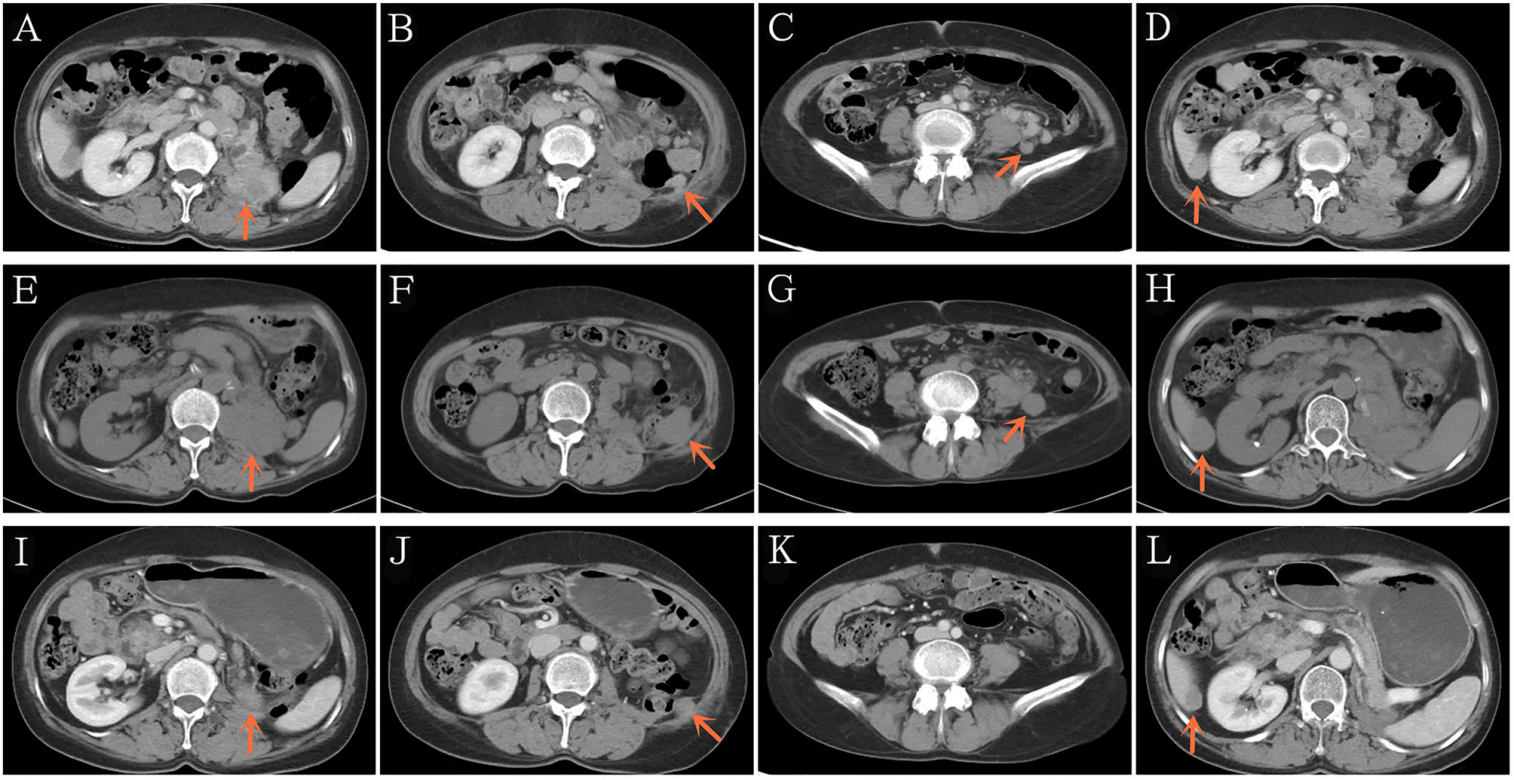
Representative computed tomography showing recurrence after 3 months of post-operative adjuvant anti-PD-1 therapy **(A–D)**. Disease continued to progress after 2 additional months of standard-dose axitinib plus anti-PD-1 therapy **(E–H)**. Significant reduction in the tumor burden after 2 additional months of low-dose axitinib plus anti-PD-1 therapy **(I–L)**. PD-1/PD-L1, Programmed death-1/programmed death ligand-1.

Three months later, she presented with a moderate gradual-onset left abdominal pain, poor appetite, and fatigue. On October 22, 2020, a whole-body enhanced CT was performed and disease relapse was apparent with evidence of newly emerged multiple metastases in the abdominal cavity and liver (**Figures 2A–D**). Following further consultation, we switched her treatment to a combination treatment regimen comprising standard-dose axitinib (5 mg, twice daily) and sintilimab (200 mg, every 3 weeks) and discharged her from hospital. After about 6 weeks, she developed binocular pain that gradually worsened and affected her eyesight more severely on the left side. On December 19, 2020, she visited a local hospital and was diagnosed with iridocyclitis. She was prescribed three eye drops: pranoprofen, tobramycin plus dexamethasone, and atropine. Simultaneously, axitinib therapy was discontinued, following the doctor’s advice. Five days later, her eye symptoms improved significantly. Due to concerns of tumor progression, she decided to restart axitinib therapy at a reduced dose of 5 mg daily. Three days later, the pain in her eyes returned; therefore, the axitinib dosage was reduced to 2.5 mg daily, after which the eye symptoms did not recur.

On December 29, 2020 (two months after treatment with axitinib plus sintilimab), she returned to our hospital for re-examination. Whole-body CT revealed progressive disease (PD), per the Response Evaluation Criteria in Solid Tumors (RECIST) 1.1 and when compared to the CT findings of October 22, 2020 (**Figures 2E–H**). These findings were supportive of the persistent abdominal pain and worsened asthenia she presented with. Since there was no particularly effective treatment regimen, and the patient had limited finances, she insisted that her previous regimen (sintilimab plus oral axitinib at a dose of 2.5 mg daily) should be continued. She was then discharged from the hospital.

We presumed that her condition would continue to gradually deteriorate, even to a life-threatening stage. However, 2 months post-discharge, on February 26, 2021, she re-visited our hospital. In the past two months, she felt that the abdominal pain has gradually relieved, her appetite and performance status have also improved. Subsequent CT revealed that the multiple metastases, including the hepatic metastases, had shrank significantly (**Figures 2I–L**), and a partial response (PR) had been achieved. To date, our patient is receiving treatment with sintilimab and axitinib at a dose of 2.5 mg daily. The most recent CT results confirmed her PR status.

## Discussion

Our patient received adjuvant treatment with anti-PD-1 therapy immediately after radical surgery. However, three months later, surveillance CT revealed multiple enhanced nodules in the abdominal cavity and liver, accompanied by clinical deterioration; this was consistent with disease relapse and resistance to anti-PD-1 therapy. Subsequently, she was administered rescue treatment with standard-dose axitinib and sintilimab for two months. However, the lesions increased significantly, and her clinical state worsened; this was assessed as PD per RECIST 1.1. This result confirmed the primary resistance to anti-PD-1 therapy and simultaneous resistance to standard-dose axitinib. After two additional months of continuous anti-PD-1 therapy and low-dose axitinib, she achieved PR, which has been observed till now. Therefore, we speculated that axitinib successfully reversed the primary resistance to anti-PD-1 therapy.

There are many studies that are consistent with our speculation: Despite the fact that the mechanisms of resistance to anti-PD-1/PD-L1 therapy are complex, and largely unknown, it is reported that the immunosuppressive tumor microenvironment (TME) plays an important role in conveying resistance, and the mechanisms include hypoxia, increased inhibitory immune cells and immune molecules, and inhibition of immune-cell trafficking and infiltration (4, 7, 8). Interestingly, evidence from numerous preclinical studies suggests that anti-angiogenic agents such as bevacizumab and vascular endothelial growth factor receptor (VEGFR) tyrosine kinase inhibitors (TKIs) including axitinib, can improve the immunosuppressive TME significantly (9–13). Directly, anti-angiogenic therapies can inhibit immunosuppressive cells and molecules, and activate anti-tumor effector immune cells. For example, axitinib can hamper myeloid-derived suppressor cells (MDSCs) by downregulating signal transducer and activator of transcription 3 (STAT3) expression in murine RCC xenografts (9), and promote immune-mediated anti-tumor activity by promoting natural killer cell recognition and degranulation in human RCC cells (10). Indirectly, anti-angiogenic therapies can promote normalization of tumor vasculature, which includes reduction in the tortuosity of tumor vessels and interstitial fluid pressure, enhancement of vessel maturation, higher pericyte coverage, and normalization of the basement membrane. Eventually, these result in an alleviation of hypoxia and an increase in tumor-infiltrating lymphocytes. Therefore, even though axitinib did not exhibit any anti-tumor effect of itself after the initial two months’ treatment, it may have assisted sintilimab in eliciting a powerful anti-tumor immune response, which resulted in subsequent tumor regression.

Given that anti-angiogenic agents are advantageous for immunotherapy, it raises an important issue: Is the dose in the drug notice optimal for them to act as immunopotentiators? Many clinical trials involving a combination of anti-angiogenic agents and anti-PD-1/PD-L1 antibodies in various types of cancers have been conducted. Now we summarized the relevant data in RCC (**Table 1**), in which anti-angiogenic agents were all administered at the dose in the drug notice. Based on clinical trial data, the objective response rates (ORRs) for the combination regimens were all significantly higher than those for single-agent treatment with multi-targeted VEGFR TKIs, such as sunitinib, sorafenib, axitinib, pazopanib, and lenvatinib. However, from a clinical perspective, it remains unclear whether anti-angiogenic agents enhance the immunotherapeutic effect of anti-PD-1/PD-L1 antibodies. Additionally, if this synergistic effect exists, it is unclear whether it has been fully exploited. As we know, except bevacizumab, both VEGFR TKIs and anti-PD-1/PD-L1 antibodies have shown a considerable single-agent efficacy for metastatic RCC, and more interestingly, the bevacizumab-based regimen was associated with the lowest ORR. Furthermore, no association has been observed between the expression of PD-L1 and outcomes in patients treated with axitinib plus pembrolizumab (16) or axitinib plus avelumab (17). Therefore, it is possible that the marked efficacy of the combination regimen was almost directly additive from that of the individual agents, or that the synergistic effect of anti-angiogenic agents on the anti-PD-1/PD-L1 antibodies in this combination regimen was limited. Furthermore, the combination regimens were often associated with severe toxicities, especially in the first three trials of pazopanil or sunitinib plus anti-PD-1 therapy (14, 15) (**Table 1** **)**. With the severe toxicities, further phase III trials have been prohibited.

**Table 1:** Summary of clinical trials evaluating the combination of PD-1/PD-L1 inhibitors plus different anti-angiogenesis agents in treatment-naïve mRCC.

Phase	Number of patients	Regimen	ORR	PFS (months)	OS (months)	Grade 3-5 AEs
I/II	20	Pazopanib+pembrolizumab (14)	20-60%	NR	NR	90%
I	33	Sunitinib+nivolumab (15)	55.0%	12.7	NR	82.0%
I	20	Pazopanib+nivolumab (15)	45.0%	7.2	27.9	70.0%
III	432	Axitinib+pembrolizumab (16)	59.3%	15.1	NR	75.8%
III	442	Axitinib+avelumab (17)	56.0%	13.8	NR	71.2%
III	454	Bevacizumab+atezolizumab (18)	37.0%	11.2	33.6	40.0%
III	355	Lenvatinib+pembrolizumab (19)	71.0%	23.9	NR	82.4%

PD-1/PD-L1, Programmed death-1/programmed death ligand-1; mRCC, metastatic renal cell carcinomas; ORR, overall response rate; PFS, progression-free survival; OS, overall survival; AEs, adverse events; NR, not reported.

**Figure 3:**
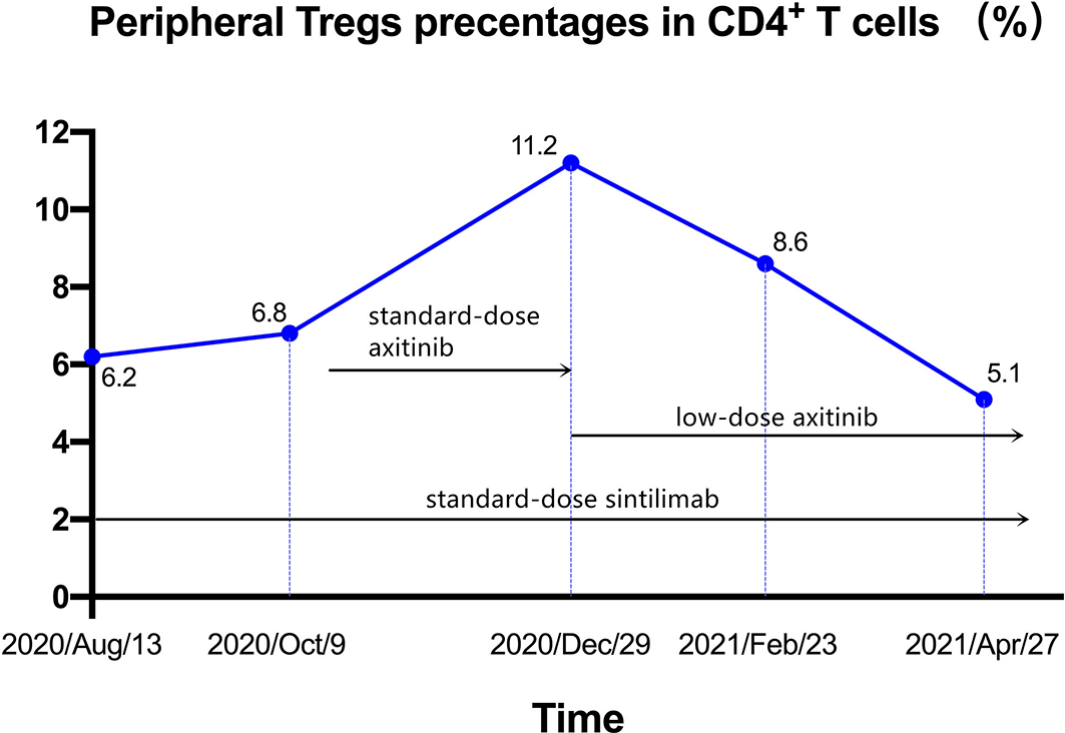
Changes of peripheral Tregs percentages in CD4^+^ T cells.

Our patient also experienced the treatment related adverse event (AE), iridocyclitis, and the dosage of axitinib was decreased to 2.5mg daily. Interestingly, the tumor remission occurred after the dose reduction, so we guess whether the dose reduction was related to the extraordinary efficacy. We retrospectively reviewed findings of the analysis of the patient’s peripheral immune cells (**Supplementary Table S1**) during her treatment course and focused on the changes in regulatory T cells (Tregs), which are reportedly detrimental to anti-tumor immunity (20, 21). As shown in **Figure 3**, before the addition of axitinib, the level of Tregs was relatively low. Two months after the addition of standard-dose axitinib, the level of Tregs increased markedly, and after the dose reduction, the level of Tregs decreased gradually, accompanied by tumor regression. The increase in levels of peripheral Tregs was likely related to standard-dose axitinib, and it was likely that there was a better synergy between low-dose axitinib and sintilimab, which induced tumor regression without affecting the Tregs. Furthermore, two studies (22, 23) of murine models (Lewis lung carcinoma and syngeneic breast cancer) demonstrated that, only low-dose anti-VEGFR2 antibody or apatinib (a novel multi-targeted VEGFR2-TKI) could maximumly alleviate hypoxia, increase the infiltration and activation of immune cells, and eventually assist anti-PD-1 therapy to maximize the anti-tumor effect in mouse models, when compared to intermediate and high doses. One explanation for this phenomenon is that the high-dose anti-angiogenic agents can lead to shorter time-windows of normalization due to rapid and excessive pruning of tumor vessels, further restricting access into the tumor parenchyma to effector T cells. Moreover, this would result in more severe hypoxia, which will further worsen the immunosuppressive TME, and thus compromise the efficacy of immunotherapy (24). Contrarily, it is possible that vascular-normalizing lower doses of anti-angiogenic agents can better alleviate hypoxia, increase the infiltration of lymphocytes, and further enhance the efficacy of immunotherapy, with fewer side-effects.

Another issue worth discussing is the iridocyclitis, which is a rare AE of anti-PD-1 therapy (25), however, iridocyclitis is not a known AE of axitinib monotherapy. Interestingly, in our patient, the aggravation and mitigation of iridocyclitis was closely related to the re-initiation and dose reduction of axitinib, and the continuous treatment with sintilimab did not cause relapse of iridocyclitis. As an immune-suppressive pathway, PD-1/PD-L1 signaling is very important to the immunity homeostasis of the body (26). Therefore, we highly suspect that axitinib induced iridocyclitis in the context of anti-PD-1 therapy and was correlated with the axitinib dosage.

There are many anti-angiogenic agents currently in clinical use. Despite inhibition of the VEGF pathway being a common characteristic of them, they also possess considerable heterogeneity. Some of these agents are monoclonal antibodies, and some are TKIs with different targets that generate different biological effects and even have unknown properties. Nevertheless, based on clinical trial data, axitinib should be a preferred option in combination with immunotherapy. The combination regimen consisting of axitinib plus pembrolizumab/avelumab has been approved as a first-line treatment option for patients with metastatic RCC. Furthermore, promising outcomes (ORR, 67.5%; progression free survival, 7.5 months) were reported in a phase I trial (27) of axitinib plus toripalimab (an anti-PD-1 antibody) in patients with advanced mucosal melanoma, who reacted poorly to anti-PD-1 monotherapy.

In conclusion, this is the first report of axitinib successfully reversing primary resistance to anti-PD-1 therapy in a patient with RCC. It’s worth noting that low-dose axitinib might be more beneficial than standard-dose axitinib in assisting immunotherapy. Given that this is a single-case report, the immunomodulatory effect of axitinib requires further investigation.

## Patient Perspective

A written informed consent for the publication has been obtained from the patient. From patient perspective, although she felt pain and weakness as the disease recurred and progressed, fortunately, she did not have too much fear and anxiety. She just insisted on treatment without paying too much attention to the effect of treatment.

## Data Availability Statement

The original contributions presented in the study are included in the article/**Supplementary Material**. Further inquiries can be directed to the corresponding authors.

## Ethics Statement

The studies involving human participants were reviewed and approved by Henan Cancer Hospital Medical Ethics Committee. The patients/participants provided their written informed consent to participate in this study.

## Author Contributions

YY collected data and wrote the manuscript. ZW and YS conceived and corrected the manuscript. QG appraised and edited the manuscript. TL and HH reviewed and edited the manuscript. All authors contributed to the article and approved the submitted version.

## Funding

This work was supported by the Henan Medical Science and Technology Research Plan (Grant No. LHGJ20190646), the Medical Science and Technology Research Project of Health Commission of Henan Province (2018010033), and the Henan Provincial Scientific and Technological Project (212102310750). The funding bodies played no role in the design of the study; the collection, analysis, and interpretation of data; or manuscript preparation.

## Conflict of Interest

The authors declare that the research was conducted in the absence of any commercial or financial relationships that could be construed as a potential conflict of interest.

## Publisher’s Note

All claims expressed in this article are solely those of the authors and do not necessarily represent those of their affiliated organizations, or those of the publisher, the editors and the reviewers. Any product that may be evaluated in this article, or claim that may be made by its manufacturer, is not guaranteed or endorsed by the publisher.

## Abbreviations

AE, Adverse event; CT, Computed tomography; MDSCs, Myeloid-derived suppressor cells; ORR, Objective response rates; PD, Progressive disease; PD-1, Programmed cell death-1; PD-L1, Programmed cell death-ligand 1; PR, Partial response; RCC, Renal cell carcinoma; RECIST, Response evaluation criteria in solid tumors; STAT3, Signal transducer and activator of transcription 3; TKIs, Tyrosine kinase inhibitors; TME, Tumor microenvironment; Tregs, Regulatory T cells; VEGF, Vascular endothelial growth factor.
